# Supplementary material for: Do Pregnancy-Induced Brain Changes Reverse? The Brain of a Mother Six Years after Parturition
Source: Brain Sci. 2021 Jan 28;11(2):168. doi: 10.3390/brainsci11020168 (PMC7912216; doi:10.3390/brainsci11020168)
Supplement: Supplementary file 1 [file brainsci-11-00168-s001.pdf]

SUPPLEMENTARY MATERIAL

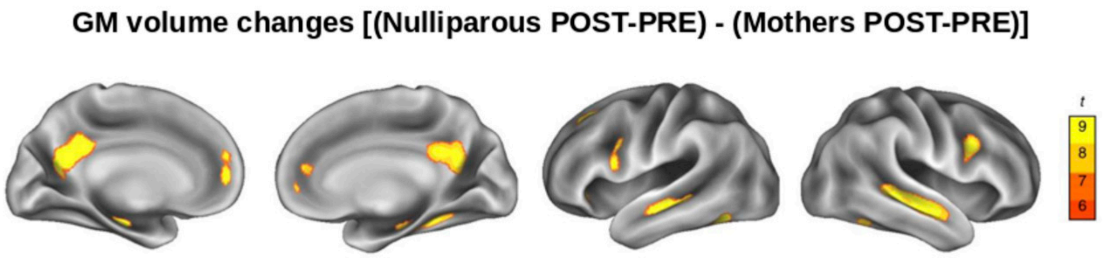

Figure S1. Regions where gray matter volume decreases more in the mothers' group (N= 25) than in the nulliparous control group (N= 20) (at a whole-brain threshold of P-value< 0.05, family-wise error (FWE)-corrected). Figure extracted from "Pregnancy leads to long-lasting changes in human brain structure" by Hoekzema et al., 2017. Reproduced with permission of Springer Nature. Abbreviations are as follows: GM= gray matter, PRE= pre-pregnancy session, POST= early postpartum session.

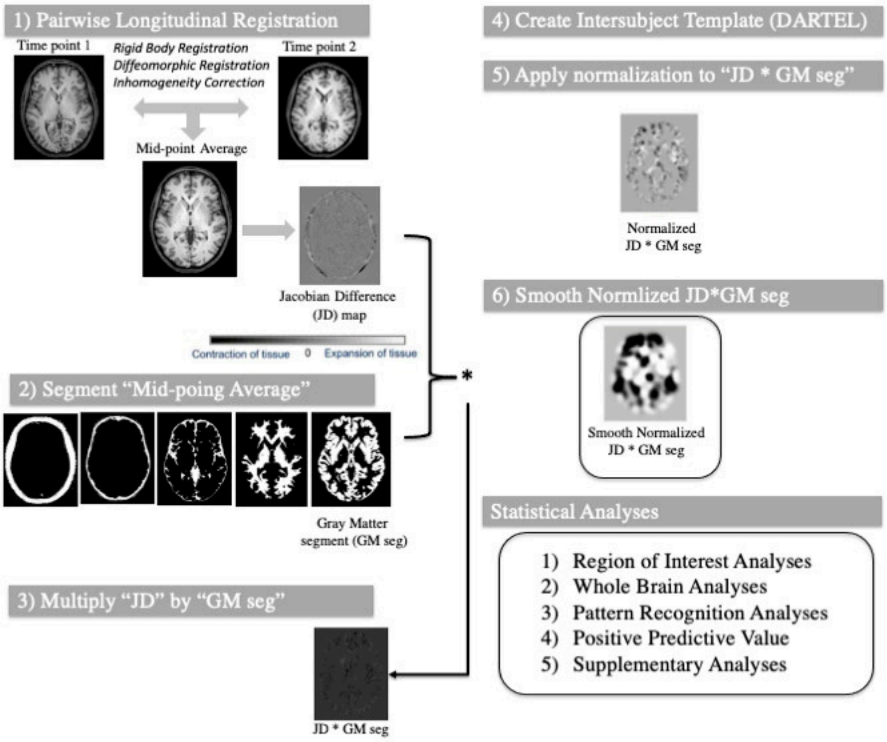

Figure S2. Image processing and statistical analysis. This figure describes the longitudinal symmetric diffeomorphic pipeline used in the study.

Supplementary Methods and Results 1

During the acquisition of the POST6y session, there was an MRI software update on the Philips scanner (from version 5.1.7 to version 5.3.1). Six participants out of twelve were scanned with a different software version. Despite maintaining constant the image reconstruction parameters, when inspecting in detail the images, we noticed that the MRI software update slightly affected the image reconstruction. In one of the software versions, there were incompatibilities between acquisition and reconstruction parameters. These incompatibilities affected the relation between the voxel size and the acquisition field of view. As a result, images with the newest software version suffered from a subtle enlargement on the phase encoding direction. The magnitude of this enlargement was estimated based on affine nine degrees of freedom registrations between the postpartum session (POST) and the six years postpartum session (POST6y). Affine registrations were performed with FSL-FLIRT between the brain-extracted images using weighting volumes to give more importance to the inner structures. The average of the 3x3 matrix determinants resulting from the affine nine of transformation was calculated. To perform the correction, we applied the diagonal transformation matrix with the scaling factor in the phase encoding direction to the images. Importantly, the same scaling factor was applied to all the subjects with the newest MRI software, thus ensuring that we maintain intrasubject variability.

The reconstruction differences between the software versions 5.1.7 and 5.3.1 were communicated to Philips so they can inform other users whose longitudinal data could also be affected.

To demonstrate that the inclusion of this correction step did not significantly change the interpretation of our findings, below we show the results --group differences-- obtained without applying the software correction.

As observed in Supplementary Figure 1, the tendencies in the mothers' group are maintained. However, in the control subjects, which were all scanned after the software update, gray matter (GM) volume reductions are closer to zero, thus accentuating and biasing both group differences (all POST6y-PRE P-values< 0.0152).

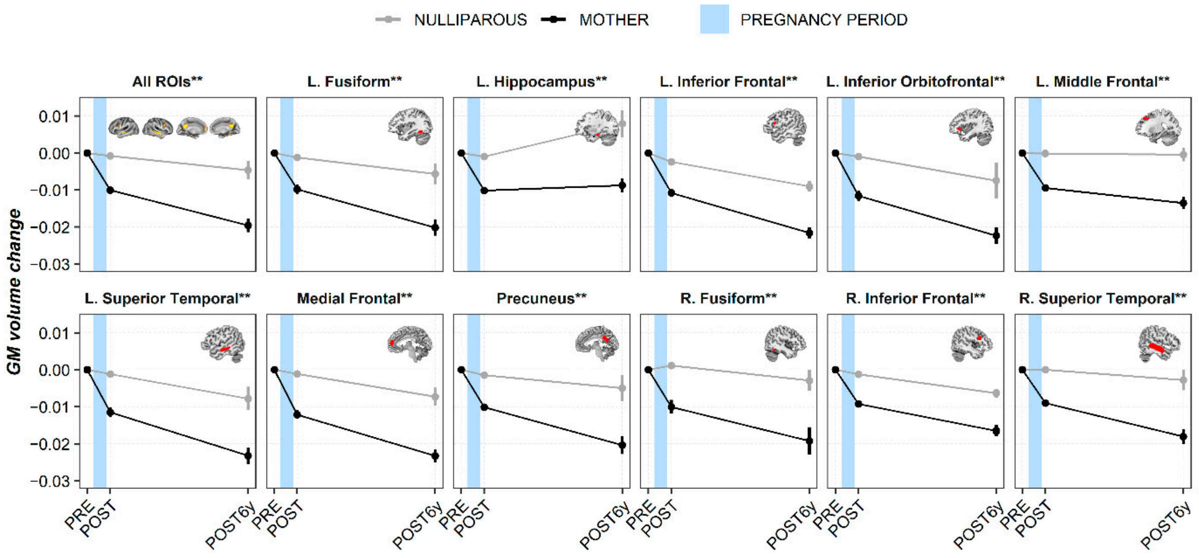

Figure S3. Gray matter volume changes for every region of interest at every time point without software correction. Results of the early postpartum session were displayed as reference values [Hoekzema et al., 2017]. Mean values (circle) with their respective standard error of the mean (vertical lines) and slopes (lines joining the

circles) are represented. Black and gray lines represent mothers and nulliparous women, respectively. The blue shadow indicates the approximated period of pregnancy. Abbreviations are as follows: GM= gray matter, L.= Left hemisphere, R.= Right hemisphere, PRE= pre-pregnancy session, POST= early postpartum session, POST6y= six years after parturition session. Asterisks indicate group differences at q< 0.05 FDR-corrected for multiple comparisons.

Supplementary Methods and Results 2

Methods:

Given our limited sample size, we calculated the Positive Predictive Value (PPV) of our main results as a post hoc analysis. The PPV is the probability that a "positive" or significant finding reflects a true effect. This probability was calculated for the between group differences in GM volume change (POST6y-PRE) in the "All ROIs" mask. This probability depends on three parameters: 1) the statistical power, which in turn depends on the effect size and sample size; 2) the threshold for statistical significance, and 3) the odds that a tested effect is a truly non-null effect among the effects being tested, also known as pre-study odds. The PPV can be estimated with the following formula:

$$PPV = ([1 - \beta] \times R) \; / \; ([1 - \beta] \times R + \alpha)$$

Where β is the probability of obtaining a false negative, $(1-\beta)$ is the statistical power, α is the probability of obtaining a false positive, and R is the pre-study odds.

We estimated the Cohen's d effect size by transforming the Mann-Whitney's U statistic ((Lenhard and Lenhard, 2016); https://www.psychometrica.de/effect_size.html), and calculated the statistical power of our test $(1-\beta)$ with the software G*power; Version 3.1.9.4, http://www.psychologie.hhu.de/arbeitsgruppen/allgemeine-psychologie-und-arbeitspsychologie/gpower.html). The following parameters were used: Test Family= t-test, Statistical test= Wilcoxon-Mann-Whitney, Tails= One, Parent distribution= min ARE, alpha error probability= 0.05, Sample size group 1= 7, Sample size group 2= 5.

The study of Hoekzema et al., 2017 is the only publication available from which to estimate our pre-study odds of the long-term effects of pregnancy. We are taking a confirmatory approach in an a priori selected ROI. Thus, the pre-study odd would be larger than 1. We have calculated the PPV as a function of different pre-study odds (from 0 to 2). To be prudent, we considered pre-study odd equals to 1, meaning that, based on previous literature, we expect the same probability for non-null effects and null effects.

Results:

The estimated Cohen's d effect size of the between-group differences in the GM volume change (POST6-PRE) of the "All ROIs" mask was 1.635, and the statistical power of the test was 0.768. The large effect size counteracted the reduced PPV commonly associated with a reduced sample size. As indicated in Supplementary Figure 2, for a pre-study odds equal to 1, the probability that our group differences in the "All ROIs" mask reflect a true effect is 0.939.

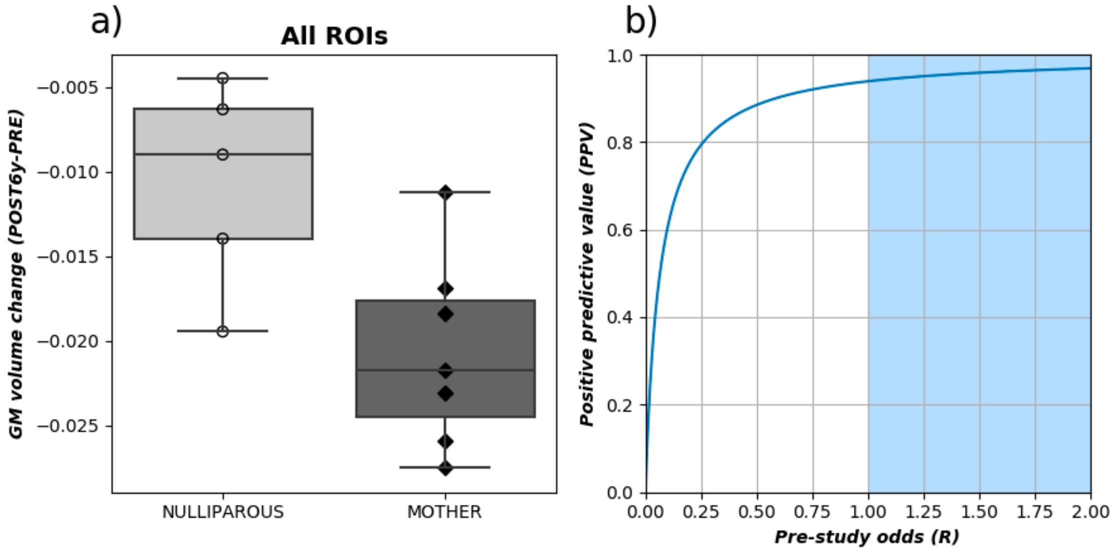

Figure S4. Positive Predictive Value (PPV) of the main results: a) Boxplot showing the gray matter (GM) volume changes between the pre-pregnancy and six years after parturition sessions in mothers and nulliparous women for the "All ROIs" mask. **b)** Graphical representation of the estimated Positive Predictive Value as a function of different pre-study odds. The considered significance level is P-value< 0.05, and the statistical power of the test is 0.768. The blue shadow indicates the values of R larger than 1. Abbreviations are as follows: PRE= pre-pregnancy session, POST6y= six years after parturition session.

Supplementary Methods and Results 3

Due to a technical problem, the radiofrequency head coil (RFHC) was replaced in three participants out of twelve. To make sure that our findings do not depend on this variable, we repeated the main analysis excluding the three participants scanned with a different RFHC.

As observed in the figure below, the patterns of GM volume changes and group differences are very similar.

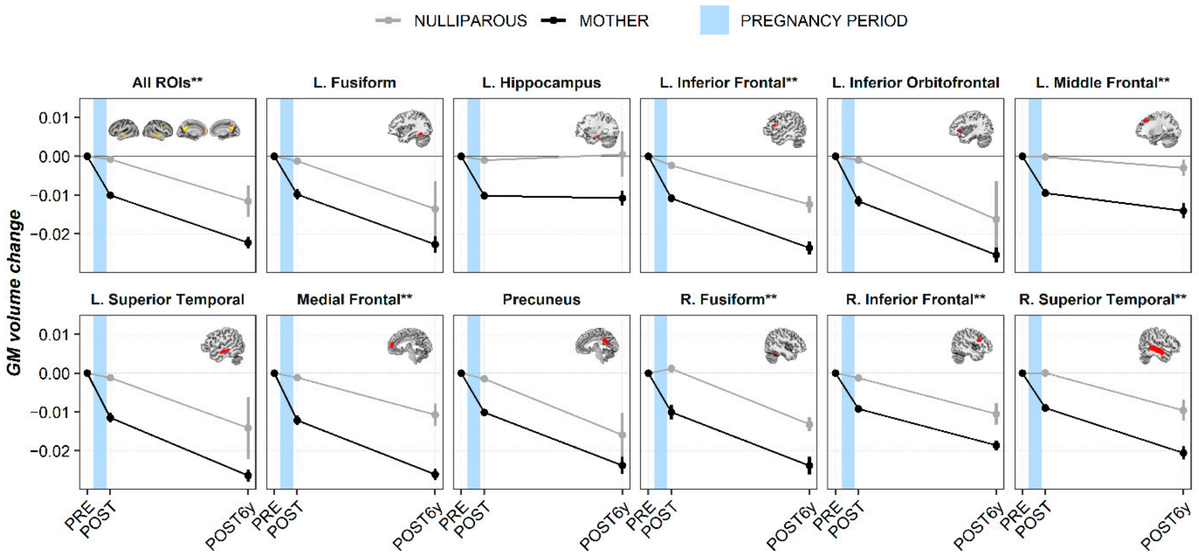

Figure S5. Gray matter volume changes for every region of interest at every time point excluding the radiofrequency head coil. Results of the early postpartum session were displayed as reference values [Hoekzema et al., 2017]. Mean values (circle) with their respective standard error of the mean (vertical lines) and slopes (lines joining the circles) are represented. Black and gray lines represent mothers and nulliparous women, respectively. The blue shadow indicates the approximated period of pregnancy. Abbreviations are as follows: GM= gray matter, L.= Left hemisphere, R.= Right hemisphere, PRE= pre-pregnancy session, POST= early postpartum session, POST6y= six years after parturition session. Asterisks indicate group differences at q< 0.05 FDR-corrected for multiple comparisons.

Table S1. Clinical and parturition data.

Group	MOTHERS			NULLIPAROUS		
**Session**	PRE	POST	POST6y	PRE	POST	POST6y
**Previous medical conditions**						
**-Depression/anxiety**	3	3	1	1	1	0
**-Thyroid disorder**	3	3	0	0	0	0
**-Anorexia**	1	1	0	0	0	0
**-Endometriosis**	1	1	1	1	1	0
**-Cholesterol/low blood glucose / hypertension**	1	1	0	1	1	1
**Postpartum Clinical information**						
**Mean Edinburgh postnatal depression scale (s.d.)**	-	7.64 (4.93)	5.43 (4.39)	-	-	-
**Clinical signs of cerebral hemorrhage**	0	0	0	-	-	-
**Duration of delivery**						
**-< 14 hours**	-	17	4	-	-	-
**->= 14 hours**	-	7	2	-	-	-
-Missing data	-	1	1	-	-	-
**Type of delivery**						
-Vaginal Delivery	-	17	4	-	-	-
-Caesarian Section	-	8	3	-	-	-

Abbreviations are as follows: PRE= pre-pregnancy session, POST= early postpartum session, POST6y= six years after parturition session, s.d.= standard deviation.

Table S2. Group differences in cognitive tests.

Measure	Mothers	NULLIPAROUS	U	P-value
	Mdn(IQR)	Mdn(IQR)		
**TAVEC** (post-pre)				
Correct	0.00 (8.00)	5.00(8.25)	121.5	0.0074
Intrusion	-1.00(2.00)	0.00(0.75)	144.5	0.237
Perseverance	0.00(5.00)	-1.50(4.50)	157.5	0.447
**WAIS Digits (post-pre)**	1.00 (3.00)	0.00 (3.75)	165.0	0.344
**N-Back total correct (post-pre)**	-1.37 (6.85)	0.00 (5.48)	138.5	0.410
**RT (post-pre)**	13.70 (25.00)	7.87 (38.33)	163.0	0.951

Comparisons of changes in the scores on the cognitive test and questionnaire data across sessions (‘PRE’ and ‘POST’ sessions) between the primiparous mothers and nulliparous control group. Shapiro-Wilk tests indicated that some of these variables did not follow a normal distribution, and therefore non-parametric two-tailed Mann-Whitney U tests were applied. Equal variances were confirmed using the non-parametric Levene’s test. There were no significant changes in performance on these measures, although it should be noted that larger sample sizes would be required to more reliably examine this type of data and reveal subtle effects. For the TAVEC, complete PRE/POST datasets are available of 23 primiparous women and 16 nulliparous control women (not all subjects participated in these tests in both sessions). For the N-back and RT test this is 22/15 and for the Digits test: 25/16. TAVEC = Test de Aprendizaje Verbal España-Complutense, based on the California Verbal Learning Test, Digits = the Digits subtest of the Wechsler Adult Intelligence Scale III, N-back = a visual 2-back test, RT = a simple visual reaction time task. Abbreviations: Mdn= Median, IQR = Interquartile range.

Table S3. Group differences in gray matter changes.

	Time point/group	Mothers		Nulliparous		Between group comparisons	
Region of Interest	Statistic	Mean (×10^{-4})	s.d. (×10^{-4})	Mean (×10^{-4})	s.d. (×10^{-4})	Wilcoxon P-value	Probability of superiority
Left Fusiform	GM jacobian POST-PRE	-98.278	62.728	-11.81	33.292	<0.001*	0.909
	Slope PRE to POST	-0.223	0.160	-0.035	0.082	<0.001*	0.899
	GM jacobian POST6y-PRE	-212.751	63.072	-118.853	91.962	0.0366*	0.829
	Slope POST to POST6y	-0.060	0.026	-0.023	0.023	0.0212	0.714
Left Hippocampus	GM jacobian POST-PRE	-97.934	53.455	-14.492	37.046	<0.001*	0.914
	Slope PRE to POST	-0.221	0.132	-0.048	0.118	<0.001*	0.857
	GM jacobian POST6y-PRE	-100.963	48.225	6.052	94.631	0.053	0.800
	Slope POST to POST6y	-0.012	0.024	0.023	0.026	0.0545	0.657
Left Inferior Frontal	GM jacobian POST-PRE	-106.493	56.186	-25.827	38.556	<0.001*	0.891
	Slope PRE to POST	-0.238	0.131	-0.068	0.099	<0.001*	0.861
	GM jacobian POST6y-PRE	-226.072	47.734	-136.134	32.271	0.0051*	0.943
	Slope POST to POST6y	-0.062	0.023	-0.048	0.009	0.1576	0.571
Left Inferior Orbitofrontal	GM jacobian POST-PRE	-111.932	73.743	-14.645	44.744	<0.001*	0.867
	Slope PRE to POST	-0.252	0.175	-0.043	0.136	<0.001*	0.830
	GM jacobian POST6y-PRE	-236.39	66.215	-142.121	128.053	0.0745	0.771
	Slope POST to POST6y	-0.060	0.032	-0.045	0.015	0.0818	0.629
Left Middle	GM jacobian POST-PRE	-90.236	55.863	-6.599	44.019	<0.001*	0.886

Frontal	Slope PRE to POST	-0.205	0.143	-0.021	0.122	<0.001*	0.865
	GM jacobian POST6y-PRE	-144.524	47.279	-45.51	33.016	0.0051*	0.943
	Slope POST to POST6y	-0.033	0.025	-0.022	0.016	0.1576	0.571
Left Superior Temporal Sulcus	GM jacobian POST-PRE	-113.173	64.433	-13.255	40.873	<0.001*	0.918
	Slope PRE to POST	-0.255	0.162	-0.044	0.111	<0.001*	0.876
	GM jacobian POST6y-PRE	-241.183	69.886	-134.437	103.79	0.053	0.800
	Slope POST to POST6y	-0.067	0.031	-0.039	0.023	0.0818	0.629
Medial Frontal (bilateral)	GM jacobian POST-PRE	-116.264	63.35	-17.466	39.078	<0.001*	0.922
	Slope PRE to POST	-0.261	0.150	-0.053	0.112	<0.001*	0.880
	GM jacobian POST6y-PRE	-246.43	49.838	-130.389	52.977	0.0025*	0.971
	Slope POST to POST6y	-0.060	0.027	-0.045	0.025	0.2061	0.543
Precuneus (bilateral)	GM jacobian POST-PRE	-98.429	43.877	-17.41	30.373	<0.001*	0.950
	Slope PRE to POST	-0.220	0.101	-0.045	0.076	<0.001*	0.937
	GM jacobian POST6y-PRE	-219.871	71.111	-133.792	88.08	0.0366*	0.829
	Slope POST to POST6y	-0.063	0.023	-0.042	0.015	0.0818	0.629
Right Fusiform	GM jacobian POST-PRE	-96.515	94.609	7.074	50.454	<0.001*	0.857
	Slope PRE to POST	-0.214	0.221	0.017	0.153	<0.001*	0.829
	GM jacobian POST6y-PRE	-211.873	91.277	-93.765	72.337	0.024*	0.857
	Slope POST to POST6y	-0.072	0.045	-0.046	0.017	0.0818	0.629
Right Inferior	GM jacobian POST-PRE	-88.172	46.388	-16.874	29.439	<0.001*	0.912

**Frontal**	**Slope PRE to POST**	-0.196	0.098	-0.042	0.075	<0.001*	0.895
	**GM jacobian POST6y-PRE**	-171.208	48.125	-105.837	35.429	0.0152*	0.886
	**Slope POST to POST6y**	-0.048	0.011	-0.036	0.009	0.0545	0.657
**Right Superior Temporal Sulcus**	**GM jacobian POST-PRE**	-88.300	49.96	-1.274	25.267	<0.001*	0.960
	**Slope PRE to POST**	-0.200	0.126	-0.006	0.075	<0.001*	0.931
	**GM jacobian POST6y-PRE**	-188.6	60.695	-77.489	57.876	0.0088*	0.914
	**Slope POST to POST6y**	-0.055	0.021	-0.023	0.019	0.0212	0.714

Asterisks indicate comparisons surviving FDR adjusted threshold at q<0.05. FDR-correction was adjusted separately for the following comparisons: 1) the GM jacobian means of the POST-PRE; 2) the GM jacobian means of the POST6y-PRE; 3) the POST-PRE slopes; and 4) the POST6y-POST slopes. Abbreviations are as follows: GM= gray matter, PRE= pre-pregnancy session, POST= early postpartum session, POST6y= six years after parturition session, s.d.= standard deviation, FDR= False Discovery Rate.
